# The Shame System Operates With High Precision

**DOI:** 10.1177/14747049231203394

**Published:** 2023-09-28

**Authors:** Alexie Leroux, Sébastien Hétu, Daniel Sznycer

**Affiliations:** 1 Department of Psychology, 5622 Université de Montréal, Montreal, QC, Canada; 2 Oklahoma Center for Evolutionary Analysis, Department of Psychology, Oklahoma State University, Stillwater, OK, USA

**Keywords:** shame, emotion, social valuation, evolutionary psychology, computation

## Abstract

Previous research indicates that the anticipatory shame an individual feels at the prospect of taking a disgraceful action closely tracks the degree to which local audiences, and even foreign audiences, devalue those individuals who take that action. This supports the proposition that the shame system (a) defends the individual against the threat of being devalued, and (b) balances the competing demands of operating effectively yet efficiently. The stimuli events used in previous research were highly variable in their perceived disgracefulness, ranging in rated shame and audience devaluation from low (e.g., missing the target in a throwing game) to high (e.g., being discovered cheating on one's spouse). But how precise is the tracking of audience devaluation by the shame system? Would shame track devaluation for events that are similarly low (or high) in disgracefulness? To answer this question, we conducted a study with participants from the United States and India. Participants were assigned, between-subjects, to one of two conditions: shame or audience devaluation. Within-subjects, participants rated three low-variation sets of 25 scenarios each, adapted from Mu, Kitayama, Han, & Gelfand (2015), which convey (a) appropriateness (e.g., yelling at a rock concert), (b) mild disgracefulness (e.g., yelling on the metro), and (c) disgracefulness (e.g., yelling in the library), all presented un-blocked, in random order. Consistent with previous research, shame tracked audience devaluation across the high-variation superset of 75 scenarios, both within and between cultures. Critically, shame tracked devaluation also within each of the three sets. The shame system operates with high precision.

## Introduction

The survival and reproduction of our human ancestors depended on the degree to which others valued the welfare of the focal individual. Being viewed positively by fellow group members was a key resource for our ancestors. Indications that the individual is able or willing to confer benefits on others cause others to attach value to the individual's welfare (Eisenbruch et al., 2016) and to be disposed to help and not harm the individual. The more valued an individual is, the more likely group members will take actions that benefit the individual and refrain from taking actions that benefit them but harm the individual. Conversely, indications that the individual is less able or willing to confer benefits on others, or less capable to defend her own interests, cause others to *devalue* the individual, and to correspondingly be less disposed to help and more disposed to harm the individual (Kurzban & Leary, 2001; see Goetz et al., 2012).

Emotions appear to be adaptations that coordinate cognitive, attentional, motivational, physiological, and behavioral mechanisms to solve complex adaptive problems faced by our ancestors (Al-Shawaf & Lewis, 2017; Cosmides & Tooby, 2000; Del Giudice, in press; Delton & Robertson, 2016; Keltner & Gross, 1999; Nesse, 1990; Sznycer et al., 2017). Evidence suggests that the emotion of shame is an adaptation that defends the individual against the threat of devaluation due to the spread of negative personal information (Durkee et al., 2019; Gilbert, 1998, 2000; Gilbert & McGuire, 1998; Landers et al., in press; Scarnier et al., 2018; Sznycer & Cohen, 2010; Tangney & Dearing, 2016).

Published findings support this *information threat theory of shame* (Sznycer et al., 2016; see also Fessler, 1999; Gilbert, 1997; Weisfeld & Dillon, 2012; for an alternative theory of shame, see: Tangney & Dearing, 2002; Tangney et al., 1996; Tracy & Robins, 2004). When being devalued, or when facing the threat of being devalued, people act in ways that seem well suited to diminish the likelihood and costs of devaluation. When the individual faces the threat of negative personal information spreading into the community, the individual engages in behavior to prevent, limit, or counteract the devaluative threat from others. When feeling shame, people produce a characteristic behavior configuration that includes head tilted down, downward gaze, slumped posture, and reduced linguistic behavior (Landers & Sznycer, 2022; Tracy et al., 2009; Weisfeld & Dillon, 2012). This behavior configuration is perceived by observers as denoting status loss by the displayer (Shariff et al., 2012; see Price & Sloman, 1987). Compared to the production of no display or other displays (e.g., anger), the shame display can elicit mollified reactions from audiences (De Jong, 1999; Keltner et al., 1997; Kemeny et al., 2004; Semin & Manstead, 1982), perhaps through signifying that the displayer will not challenge the reduction in status or esteem that the newfound information merits (Fessler, 1999; Gilbert, 1997; Weisfeld & Dillon, 2012). Under shame, cortisol level increases (Gruenwald et al., 2004; Lewis & Ramsay, 2002). Under shame, people withdraw (Keltner & Harker, 1998), hide, conceal incriminating information (Sznycer et al., 2015), appease (Keltner et al., 1997), and engage in altruistic behavior (Declerck et al., 2014; de Hooge et al., 2008; Tenhouten, 2017). Shamed individuals sometimes aggress against others (Elison et al., 2014; Tangney, Wagner, Fletcher et al., 1992), perhaps because aggression, though socially undesirable, is a means to bargain for better treatment (Sell et al., 2009).

The shame system appears to be mobilized not only reactively, when the actor knows that others have devalued her, but also anticipatorily, before the actor takes a discrediting act. By hypothesis (Sznycer et al., 2016), the anticipatory feeling of shame indexes the payoff from others’ devaluation (e.g., the cost you will incur from your coworker if you eat the last slices of pizza and don’t leave her any, discounted by the probability that she will find out). Added to the estimated personal payoff (e.g., the benefit you will derive from eating the pizza), the devaluation payoff determines in part what course of action the individual will take. Thus, the anticipatory feeling of shame can lead to eschewing actions or activities that others devalue, or to taking actions that others devalue but with added precautions (e.g., secrecy).

To operate cost-effectively, the shame system must be properly calibrated to the magnitude of the devaluative threat on an event-by-event basis. The under-activation of shame relative to a devaluative threat would fail to counteract part of that threat. Conversely, the over-activation of shame would lead to excessive diffidence and missed opportunities. But if well-engineered, the shame system would balance the conflicting demands of effectiveness and economy. This has led to the hypothesis that shame is generally mobilized to a degree that is just right: (a) it accurately registers (or forecasts) the magnitude of the present devaluative threat, and (b) activates in proportion to that magnitude. Thus, according to the information threat theory, what the shame system tracks (in order to minimize or counteract) is (the actor's estimate of) other people's evaluations. In contrast, according to an alternative theory of shame (attributional theory; Tangney et al., 2007) what shame tracks is the actor's own evaluations of, or attributions about, her shortfalls.

Consistent with the information threat theory, previous studies have shown that the intensity of anticipatory shame people feel when they imagine themselves taking an action that others devalue is closely associated with the magnitude of devaluation that local audiences, and even foreign audiences, direct at individuals who take that action (Cohen et al., 2020; Durkee et al., 2019; Sznycer & Cohen, 2021; Sznycer & Patrick, 2020; Sznycer et al., 2016; Sznycer et al., 2018). For example, the more American participants devalue an individual if that individual takes a certain disgraceful action, the more (other) American participants, and even Israeli and Indian participants, report anticipatory shame when they imagine themselves taking that action. This pattern of findings was observed across industrial societies (e.g., Sznycer et al., 2016), across traditional small-scale societies (Sznycer et al., 2018), and over millennia (Sznycer & Patrick, 2020). Further, the association with audience devaluation is specific to shame; it doesn’t generalize to other negatively valenced emotions such as sadness or anxiety (Sznycer et al., 2016).

The stimuli events used in previous research were highly variable in their perceived disgracefulness. These events ranged in ratings of shame and devaluation from low (e.g., missing the target in a throwing game) to high (e.g., being discovered cheating on one's spouse). Certainly, the shame system is precise enough to have produced the positive associations described above. But how precise is the shame system? Do positive associations between shame and devaluation depend on there being high variation in how disgraceful the events are—the kinds of stimuli events that were studied previously? Or will shame track audience devaluation even when the stimuli events vary little in their disgracefulness?

Here, we aim to answer this question. We conducted a study with participants from the United States and India. Participants in each country were randomly assigned, between-subjects, to one of two conditions: a *shame* condition or a *devaluation* condition. Within-subjects, participants rated 75 scenarios constructed to convey (a) appropriateness (e.g., yelling at a rock concert; 25 scenarios), (b) mild disgracefulness (e.g., yelling on the metro; 25 scenarios), and (c) disgracefulness (e.g., yelling in the library; 25 scenarios). This design allows us to determine whether the tracking of devaluation by shame is observed, within and between cultures: (a) in the high-variation superset of scenarios, across disgracefulness levels, and, critically, (b) within each of the three low-variation sets of scenarios.

## Study

We adapted the social norm violation task developed by Mu et al. (2015). The adapted task consists of 25 actions (e.g., yelling) presented in three different social contexts: *appropriate* (e.g., yelling at a rock concert), *mildly disgraceful* (e.g., yelling on the metro), and *disgraceful* (e.g., yelling in the library). From each action-context combination (e.g., yelling on the metro), we generated two sets of stimuli: *shame* stimuli, wherein the event is described as being true of the participant (e.g., “I am on the metro yelling”), and *devaluation* stimuli, wherein the event is described as being true of an individual other than the participant (e.g., “Steven is on the metro yelling”), corresponding to our two conditions. Thus, we created 150 scenarios (25 actions × 3 contexts × 2 conditions). The scenarios in the shame and devaluation conditions were semantically similar; the main difference is the perspective from which the scenarios were described. The scenarios of the shame condition and the devaluation condition are displayed in Tables S1 and S2.

In a between-subjects design, participants were assigned randomly to one of two conditions: devaluation condition or shame condition. Participants in the devaluation condition were asked to indicate, for each of the 75 scenarios involving another individual (e.g., “Steven is on the metro yelling”), how negatively they would view that individual if that individual were in that situation; they answered using a Likert scales ranging from 1 (“I wouldn’t view them negatively at all”) to 7 (“I’d view them very negatively”). These ratings indicate the degree to which members of a given population would devalue specific actions in different contexts. Participants in the shame condition were asked to indicate, for each of the 75 scenarios (e.g., “I am on the metro yelling”), how much shame they would feel if they were in that situation. They answered using Likert scales ranging from 1 (“no shame et al.”) to 7 (“a lot of shame”).

## Methods

### Participants

We collected data from 88 participants in the United States. We removed 12 participants from analyses due to failure to complete the study or failure to correctly answer an attention check. Thus, the effective sample size in the US is 76 participants (45 females) (age: mean: 40 y; *SD*: 12 y). We collected data from 73 participants in India. We removed 11 participants from analyses due to failure to correctly answer an attention check. Thus, the effective sample size in India is 62 participants (age: mean: 29 y; *SD*: 5 y). We collected data with Amazon Mechanical Turk. The Internal Review Boards of the Université de Montréal and the Université du Québec à Trois-Rivières approved this study. We paid participants $0.25 for completing the study.

### Materials and Procedure

In the original task by Mu et al. (2015), participants rated 102 scenarios resulting from the combination of 34 actions (e.g., yelling) and three different contexts per action: *appropriate* (e.g., Steve is at a rock concert. He is yelling), *mild disgracefulness* (e.g., Steve is on the metro. He is yelling), and *disgraceful* (e.g., Steve is in the library. He is yelling)—termed by Mu et al. “appropriate,” “weak violation,” and “strong violation,” respectively. Taking Mu et al.'s (2015) 102 scenarios as our point of departure, we created 204 scenarios: 102 shame scenarios, and 102 devaluation scenarios. We conducted the study in English in the United States and India.

### Pilot Study

To find out whether participants perceived the disgracefulness of the scenarios in a way that matches Mu et al.'s design, we conducted a pilot test of the 204 scenarios on an American sample (*N* = 103). Participants in the shame condition (*N* = 55) rated, for each of the 102 shame scenarios, how much shame they would feel if they imagined themselves in those situations. They used 7-point Likert scales: 1: “no shame at all”; 7: “a lot of shame.” Participants in the devaluation condition (*N* = 48) rated, for each of the 102 devaluation scenarios, how negatively they would view the individual in the scenario. They used 7-point Likert scales: 1: “I wouldn’t view them negatively at all”; 7: “I’d view them very negatively.”

We conducted paired samples t-tests on the 34 triads of scenarios. Based on these, we discarded nine triads (27 shame scenarios; 27 devaluation scenarios) because the ordinal differences were not as expected or because the ordinal differences, though in the expected direction, were not statistically significant. The resulting set of 25 triads of scenarios (75 shame scenarios; 75 devaluation scenarios) constituted the stimuli of our study.

Next, we describe the results of the main study conducted with the definitive stimuli (25 triads of scenarios).

## Results

### Preliminary Analyses

To evaluate whether shame tracks devaluation with high precision, we used as stimuli three sets of 25 scenarios (*appropriate* set; *mildly* disgraceful set; *disgraceful* set), adapted from Mu et al. (2015). We did so on the expectations that, by stimuli design, each set of scenarios would yield ratings of devaluation and shame (1) *less* variable than the ratings of devaluation and shame observed in previous research, and (2) lower (or higher) than the next set. Preliminary analyses indicate that stimuli features (1) and (2) were not only assumed, based on how the stimuli were constructed, but also actually observed in the data.

**Figure 1.**
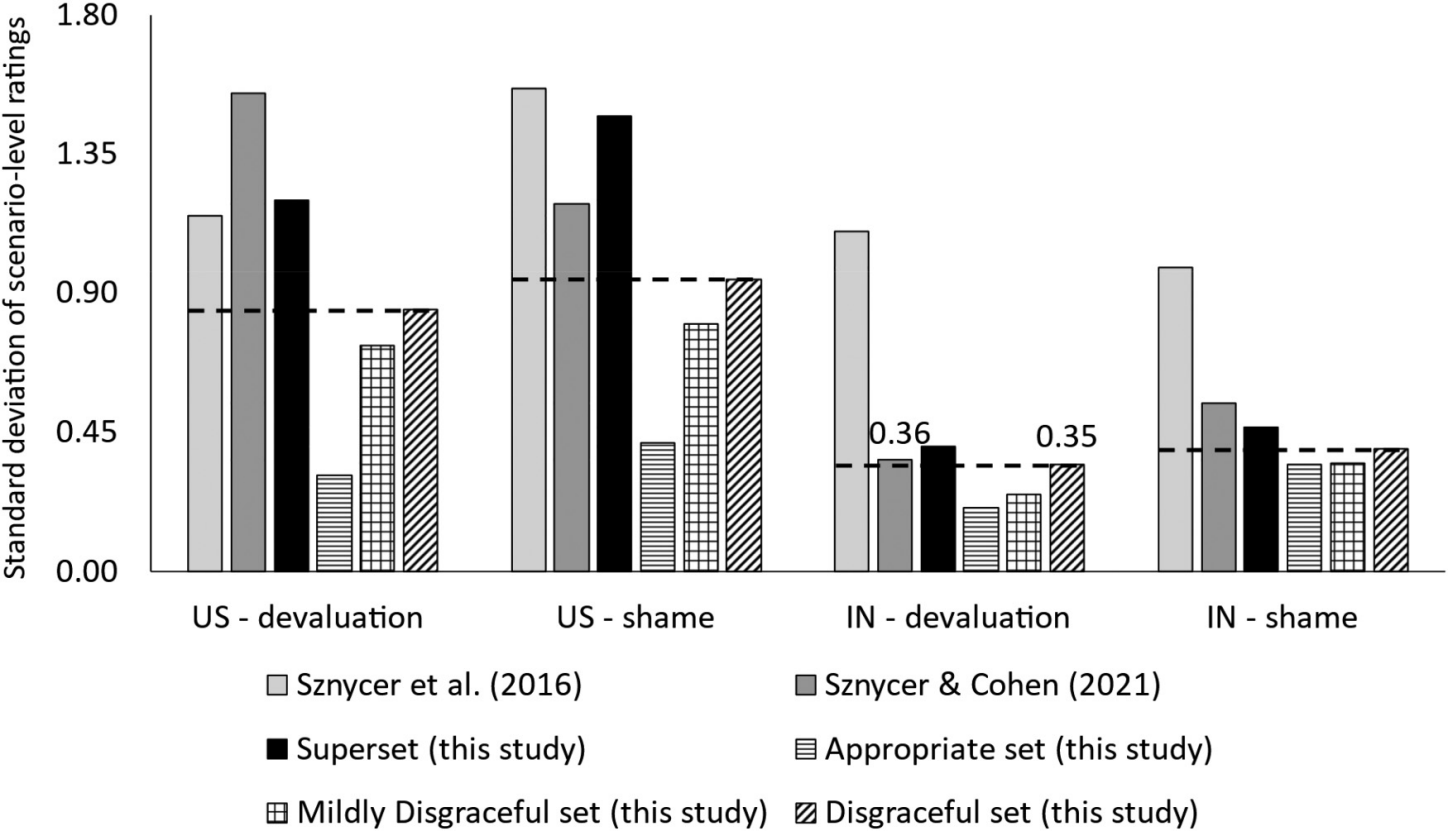
Variation in scenario-level ratings in the three sets of scenarios, compared to the variation observed in previous research. Note: displayed are the standard deviations across scenario-level (means of) ratings by condition, country of participant, and dataset. US: participants from the United States; IN: participants from India. We contrast the results of the present study against the results of two previous studies with similar characteristics: Sznycer et al. (2016), and Sznycer and Cohen (2021; data from the *shame feeling* condition). In all cases, ratings were given on 1–7 Likert scales. Number of scenarios (and scenario-level ratings): Sznycer et al. (2016): 29; Sznycer and Cohen (2021; data from the *shame feeling* condition): 27; *appropriate* set (this study): 25; *mildly disgraceful* set (this study): 25; *disgraceful* set (this study): 25; superset (this study): 75. Patterned bars indicate the standard deviations corresponding to the three sets of scenarios that constitute the focus of the present study; full bars indicate the standard deviations corresponding to the two previous studies—as well as the superset of 75 scenarios of the present study, which is also expected to yield devaluation and shame ratings more variable than the ratings observed in each constituent set. The dotted line, included for reference, indicates the highest standard deviation of any of the three set of scenarios (the *disgraceful* set in all cases, as it turned out). For each condition and each country, the highest standard deviation of any of the three sets of scenarios in the present study was always lower than the lowest standard deviation in any of the previous studies, and was also lower than the standard deviation of the superset of 75 scenarios of the present study. In sum, consistent with the way Mu et al. (2015) constructed the scenarios, there was little variation in ratings of devaluation and shame in each of the three sets of scenarios of the present study—compared to the stimuli of previous studies and the superset of 75 scenarios of the present study.

Preliminary result # 1: Ratings of devaluation and shame were less variable in each of the three sets of scenarios than in the sets of scenarios used in previous research (and in the superset of 75 scenarios of the present study). We contrast the variation observed in the present study with the variation observed in two previous studies with similar characteristics: Sznycer et al. (2016), and Sznycer and Cohen (2021; data from the *shame feeling* condition). As in the present study, in both of the previous studies, MTurk participants from the US and India rated each of several scenarios on shame (if the disgraceful event were true of the participant) and devaluation (if the disgraceful event were true of someone else) using 1–7 Likert scales. Also, as in the present study, in both of the previous studies, condition (shame vs. devaluation) was a between-subjects variable whereas scenario was a within-subjects variable. Figure 1 displays how much variation there is, in standard deviation units, across the scenario-level means of devaluation and shame ratings, by study and by country of participant. Patterned bars indicate the standard deviations corresponding to the three sets of scenarios that constitute the focus of the present study; full bars indicate the standard deviations corresponding to the two previous studies (and the superset of 75 scenarios of the present study, which is also expected to yield devaluation and shame ratings more variable than the ratings observed in each constituent set). As Figure 1 indicates, across conditions (devaluation, shame) and countries of participants (US, India), the standard deviations corresponding to the three sets of scenarios were lower than the standard deviations corresponding to the two previous studies and the superset of 75 scenarios of the present study. Indeed, for each condition and each country, the *highest* standard deviation of any of the three sets of scenarios in the present study (the *disgraceful* set in all cases, as it turned out) was always lower than the *lowest* standard deviation in any of the previous studies, and was also lower than the standard deviation of the superset of 75 scenarios of the present study. In sum, consistent with the way Mu et al. (2015) constructed the scenarios, there was little variation in ratings of devaluation and shame in each of the three sets of scenarios of the present study compared to the stimuli of previous studies and the superset of 75 scenarios of the present study.

Preliminary result # 2: Ratings of devaluation and ratings of shame were lowest in the *appropriate* set, intermediate in the *mildly disgraceful* set, and highest in the *disgraceful* set. We report these in turn.

Ratings of devaluation were lowest in the *appropriate* set, intermediate in the *mildly disgraceful* set, and highest in the *disgraceful* set. A repeated measures ANOVA indicated that in the United States the mean intensity of devaluation differed significantly across the three sets: *F*(2, 48) = 169.51, *p* = 10^−20^, *η*_p_^2^ = .88. Post-hoc analyses with Bonferroni correction indicated that devaluation for the scenarios in the *disgraceful* set (pooled mean = 4.39) was significantly higher than devaluation for the scenarios in the *mildly disgraceful* set (pooled mean = 3.09) and the *appropriate* set (pooled mean = 1.95); in turn, devaluation for the scenarios in the *mildly disgraceful* set was significantly higher than devaluation for the scenarios in the *appropriate* set (all *p*s < .001). Similarly, a repeated measures ANOVA indicated that in India the mean intensity of devaluation differed significantly across the three sets: *F*(2, 48) = 54.29, *p* = 10^−12^, *η*_p_^2^ = .69. Post-hoc analyses with Bonferroni correction indicated that devaluation for the scenarios in the *disgraceful* set (pooled mean = 4.97) was significantly higher than devaluation for the scenarios in the *mildly disgraceful* set (pooled mean = 4.48) and the *appropriate* set (pooled mean = 4.25); in turn, devaluation for the scenarios in the *mildly disgraceful* set was significantly higher than devaluation for the scenarios in the *appropriate* set (all *p*s < .001).

Ratings of shame too were lowest in the *appropriate* set, intermediate in the *mildly disgraceful* set, and highest in the *disgraceful* set. A repeated measures ANOVA indicated that in the United States the mean intensity of shame differed significantly across the three sets: *F*(2, 48) = 168.20, *p* = 10^−21^, *η*_p_^2^ = .88. Post-hoc analyses with Bonferroni correction indicated that shame for the scenarios in the *disgraceful* set (pooled mean = 4.83) was significantly higher than shame for the scenarios in the *mildly disgraceful* set (pooled mean = 3.20) and the *appropriate* set (pooled mean = 1.75); in turn, shame for the scenarios in the *mildly disgraceful* set was significantly higher than shame for the scenarios in the *appropriate* set (all *p*s < .001). Similarly, a repeated measures ANOVA indicated that in India the mean intensity of shame differed significantly across the three sets: *F*(1.58, 38.00) = 38.06, *p* = 10^−8^, *η*_p_^2^ = .61. Post-hoc analyses with Bonferroni correction indicated that shame for the scenarios in the *disgraceful* set (pooled mean = 4.87) was significantly higher than shame for the scenarios in the *mildly disgraceful* set (pooled mean = 4.57) and the *appropriate* set (pooled mean = 4.15); in turn, shame for the scenarios in the *mildly disgraceful* set was significantly higher than shame for the scenarios in the *appropriate* set (all *p*s < .01).

In sum, the observed distributions of shame and devaluation ratings indicate that the present stimuli are appropriate (a) to evaluate whether shame tracks devaluation with high precision (i.e., even when perceived disgracefulness varies little from one event to the next) (b) along a gradient of disgracefulness (low, medium, high disgracefulness). Next, we report the focal results.

### Within-Country Results

First, we report the results within each country. The scenarios and descriptive statistics are displayed in Table S3.

Do participants within a country agree with one another about how much shame they would feel when they imagine themselves performing the actions described in the scenarios? Yes. In the United States, participants agreed with one another about the degree to which they would feel shame when they imagined themselves performing the actions presented in the scenarios: In the superset of 75 shame scenarios (ICC (2,37) = .97, *p* < .001), as well as in each set of 25 shame scenarios: the *appropriate* set (ICC (2,37) = .84, *p* < .001), the *mildly disgraceful* set (ICC (2,37) = .90, *p* < .001), and the *disgraceful* set (ICC (2,37) = .93, *p* < .001). In India too participants agreed with one another about the degree to which they would feel shame when they imagined themselves performing the actions presented in the scenarios: In the superset of 75 shame scenarios (ICC (2,26) = .69, *p* < .001), as well as in each set of 25 shame scenarios: the *appropriate* set (ICC (2,26) = .58, *p* < .001), the *mildly disgraceful* set (ICC (2,26) = .54, *p* < .01), and the *disgraceful* set (ICC (2,26) = .57, *p* < .001).

Do participants within a country agree with one another about how negatively they would evaluate a target individual who performs the actions described in the scenarios? In general, yes. In the United States, participants agreed with one another about the degree to which they would devalue a target who performed the actions described in the scenarios: In the superset of 75 devaluation scenarios (ICC (2,39) = .96, *p* < .001), as well as in each set of 25 devaluation scenarios: the *appropriate* set (ICC (2,39) = .80, *p* < .001), the *mildly disgraceful* set (ICC (2,39) = .91, *p* < .001), and the *disgraceful* set (ICC (2,39) = .92, *p* < .001). In India, participants agreed with one another about the degree to which they would devalue a target who performed the actions described in the scenarios: In the superset of 75 devaluation scenarios (ICC (2,36) = .68, *p* < .001) and in the *disgraceful* set of 25 devaluation scenarios (ICC (2,36) = .53, *p* < .01), but not in the *appropriate* set (ICC (2,36) = .19, *p* = .20) or the *mildly disgraceful* set (ICC (2,36) = .256, *p* = .13).

**Figure 2.**
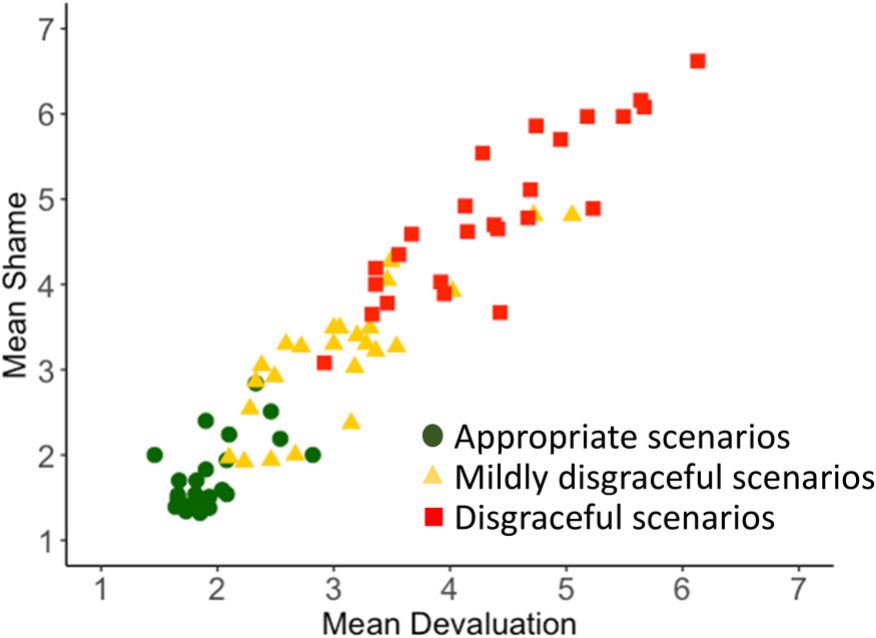
Scatterplot: shame as a function of devaluation. United States sample.

**Figure 3.**
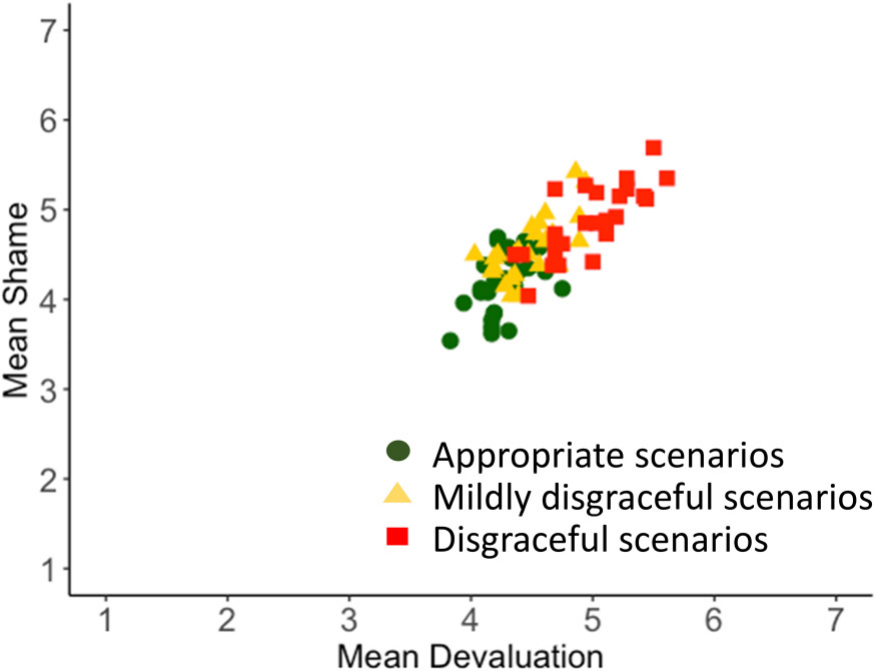
Scatterplot: shame as a function of devaluation. India sample.

Does audience devaluation predict feelings of shame? More specifically, does the intensity of others’ devaluation predict the intensity of shame that participants would feel if they performed the actions described in the scenarios? Yes. We computed mean shame ratings and mean devaluation ratings for each of the 75 shame scenarios and 75 devaluation scenarios, respectively. In the (paired) superset of 75 scenarios, shame and devaluation were strongly correlated with each other; in the United States (*r*(73) = .95, *p* = 10^−39^) and in India (*r*(73) = .80, *p* = 10^−16^) (Table 1, Figures 2 and 3). This replicates previous findings indicating that shame closely tracks audience devaluation when there is high variation in the disgracefulness of the stimuli events (Cohen et al., 2020; Durkee et al., 2019; Sznycer & Cohen, 2021; Sznycer & Patrick, 2020; Sznycer et al., 2016; Sznycer et al., 2018).

**Table 1.** Correlations Between Shame and Devaluation Within- and Between-Countries, by Sets of Scenarios.

		Devaluation	
	*Supersets of 75 scenarios*		
		US	India
	US	.95***	.79***
	India	.67***	.80***
	Sets of 25 *appropriate* scenarios		
		US	India
**Shame**	US	.57**	.30
	India	.72***	.46*
	Sets of 25 *mildly disgraceful* scenarios		
		US	India
	US	.84***	.37†
	India	.48*	.69***
	Sets of 25 *disgraceful* scenarios		
		US	India
	US	.88***	.57**
	India	.17	.77***

*Note*. Coefficients are Pearson's *r*s.

† .05 ≤ *p* ≤ .10, * *p* < .05, ** *p* < .01, *** *p* < .001.

However, the central question here is: Does shame track audience devaluation when there is *low* variation in the disgracefulness of the stimuli events? Yes. In the Unites States, shame and devaluation were correlated with each other also within each (paired) set of 25 scenarios: within the *appropriate* set (*r*(23) = .57, *p* = .003); within the *mildly disgraceful* set (*r*(23) = .84, *p* = 10^−6^); and within the *disgraceful* set (*r*(23) = .88, *p* = 10^−8^). In India too, shame and devaluation were correlated with each other also within each (paired) set of 25 scenarios: within the *appropriate* set (*r*(23) = .46, *p* = .022); within the *mildly disgraceful* set (*r*(23) = .69, *p* = .0001); and within the *disgraceful* set (*r*(23) = .77, *p* = 10^−5^). Note that the shame and devaluation ratings were given by different participants. Therefore, these correlations cannot be caused by participants matching their shame and devaluation ratings.

### Between-Country Results

From a theoretical standpoint, the shame system should be sensitive to the evaluations of those individuals whose actions would impact the actor's welfare—local audiences. Similarly, from an empirical standpoint, some acts and traits that elicit devaluation and shame in some places, times, or communities do not elicit devaluation or shame in other places, times, or communities. For example, eating with the left hand is discrediting in India but not in the United States. Nevertheless, if there are regularities in the structure and content of social-evaluative psychology and the shame system, there might be cross-cultural regularities in shame, devaluation, and the shame–devaluation link. To explore this possibility, we conducted the following analyses.

#### Is Devaluation in one Country Associated With Devaluation in the Other Country?

Yes. To evaluate agreement in devaluation between the American and Indian participants, we computed the correlations across mean devaluation ratings given by the American and Indian participants. For the superset of 75 devaluation scenarios, American and Indian participants agreed about the degree to which a situation would elicit devaluation (*r*(73) = .77, *p* = 10^−15^). Importantly, this cross-cultural agreement in devaluation was observed also within the *mildly disgraceful* set (*r*(23) = .51, *p* = .009) and the *disgraceful* set (*r*(23) = .44, *p* = .029), though not in the *appropriate* set (*r*(23) = .22, *p* = .29).

#### Is Shame in one Country Associated With Shame in the Other Country?

Yes. To evaluate agreement in shame between the American and Indian participants, we computed the correlations across mean shame ratings given by the American and Indian participants. For the superset of 75 shame scenarios, American and Indian participants agreed about the degree to which a situation would elicit shame in them (*r*(73) = .72, *p* = 10^−12^). Importantly, this cross-cultural agreement in shame was observed also within the *appropriate* set (*r*(23) = .58, *p* = .002), the *mildly disgraceful* set (*r*(23) = .43, *p* = .033), and the *disgraceful* set (*r*(23) = .43, *p* = .032).

#### Does Shame in one Country Track Devaluation in the Other Country?

Yes. For the superset of 75 scenarios, shame in the United States tracked devaluation in India (*r*(73) = .79, *p* = 10^−16^), and shame in India tracked devaluation in the United States (*r*(73) = .67, *p* = 10^−10^). The more a scenario elicited devaluation in one country, the more that scenario elicited shame in the other country. Strikingly, cross-cultural correspondences between shame and devaluation were observed also within each (paired) set of 25 scenarios—always in the expected direction, though only sometimes significantly. Within the *appropriate* set, shame in India tracked devaluation in the United States (*r*(23) = .72, *p* = 10^−4^); however, shame in the United States failed to significantly track devaluation in India (*r*(23) = .30, *p* = .14). Within the *mildly disgraceful* set, shame in India tracked devaluation in the United States (*r*(23) = .48, *p* = .016); shame in the United States marginally tracked devaluation in India (*r*(23) = .37, *p* = .067). Within the *disgraceful* set, shame in the United States tracked devaluation in India (*r*(23) = .57, *p* = .003); shame in India failed to significantly track devaluation in the United States (*r*(23) = .17, *p* = .42). See Table 1.

## Discussion

Previous findings indicate that anticipatory shame is elicited in proportion to the magnitude of the devaluative threat (Cohen et al., 2020; Durkee et al., 2019; Sznycer & Lukaszewski, 2021; Sznycer et al., 2020; Tangney & Dearing, 2016; Tangney et al., 2018; see also Sznycer et al., 2019; Sznycer, 2022). However, the stimuli events used in previous studies varied greatly in their perceived disgracefulness within study. Thus, previous studies were not well suited to determine whether shame tracks devaluation with precision. Does anticipatory shame track devaluation even when the stimuli events vary little in their perceived disgracefulness? The present data indicate that it does. Within each set of scenarios—*appropriate*, *mildly disgraceful*, and *disgraceful*—shame tracked the devaluation of local audiences, both in the United States and in India, with large to very large effect sizes. Strikingly, within each set of scenarios, shame in the United States and India tended to track the devaluation of audiences *in the other* country. These cross-cultural matches may owe to the fact that the original stimuli by Mu et al. (2015) refer to concepts such as “metro” and “library” which are culturally meaningful in the United States and India, though not in other populations. Therefore, these cross-cultural matches may not replicate in, for example, traditional small-scale societies—although stimuli phrased at a higher level of abstraction (e.g., being stingy, being lazy) can and does yield shame–devaluation matches across small-scale societies (Sznycer et al., 2018). The present finding that shame tracks local audiences more highly than it tracks foreign audiences is consistent with previous findings (e.g., Sznycer et al., 2016) as well as with the hypothesis that shame is tuned to the evaluations of those individuals whose actions impact the actor's welfare—other individuals in one's local social ecology.

The tracking of audience devaluation by the shame system bears the stamp of natural selection, because (a) it enables the shame system to balance the conflicting demands of effectiveness and economy, and (b) it is improbably well suited to solve the adaptive problem of information-triggered devaluation; in addition, (c) shame's tracking of local devaluation has been observed in every population studied to date, including industrial societies and traditional small-scale societies (Sznycer & Cohen, 2021; Sznycer et al., 2016; Sznycer et al., 2018; see also Sznycer & Patrick, 2020), so this appears to be a species-typical design feature rather than an adaptation to local conditions.

Theoretically, the maximum precision attainable by the shame system, in absolute terms and on a context-to-context basis, would be set where the incremental benefits of higher discriminativeness are offset by the computational costs of attaining such discriminativeness. Selection for minimizing the costs of being devalued—the costs of being abandoned, betrayed, punished, ostracized, or killed in a socially sanctioned execution—would have been strong (see von Rueden & Jaeggi, 2016; Wrangham, 2018), and therefore capable of crafting highly precise (and effective) adaptations. The fact that shame tracked devaluation with substantial effect sizes *even within the set of scenarios with the least variation in disgracefulness* (the *appropriate* set) suggests that the shame system is highly precise. At the limit, shame might track just noticeable differences in audience devaluation. Future research might elucidate the upper boundary of precision, how much precision is attainable in different regions of the devaluation gradient (e.g., minor vs. moderate disapproval), and how different cues of being devalued (e.g., visual, linguistic) are processed and transduced into underlying representations of social devaluation.

Although a growing body of research indicates robust quantitative correspondences between shame and audience devaluation, the problem of how to limit the cost of devaluation in different contexts has received less attention. But solving this problem is critical too because a shame response that is of the wrong type is futile or counterproductive even when it precisely matches in intensity the intensity of audience devaluation. For example, although in some contexts appeasing has beneficial effects for the shamed individual (Keltner et al., 1997), in other contexts appeasing may *reveal* your culpability and *cause* devaluation—for instance, when you ascertain that, prior to the appeasement, others have *not* registered the commission of a discrediting act or the identity of the offender (De Jong et al., 2003; Landers & Sznycer, 2022). More research is needed to characterize the menu of outputs available to the shame system, the mappings between inputs and outputs, and the decision architecture that tailors fine-grained behavioral decisions to the current situation (Lukaszewski, 2021; Lukaszewski et al., 2020).

The present correlational data raise the question: Is audience devaluation the cause of shame (of its evolution and of its moment-to-moment activation), as we hypothesize here, or does shame cause others to devalue the (shamed) individual? We believe devaluation causes shame and not the other way around. Experimental manipulations of devaluative threat reliably mobilize shame in the individual (Dickerson et al., 2009; Landers et al., forthcoming; Modigliani, 1971; Robertson et al., 2018; Scarnier et al., 2009; Smith et al., 2002), as predicted by the information threat theory of shame. In contrast, displays of shame *diminish* an audience's devaluative response when the audience and the individual have joint attention regarding a discrediting act (De Jong, 1999; Keltner et al., 1997; Semin & Manstead, 1982). As noted above, by acting ashamed you may give yourself away and cause others to devalue you (De Jong et al., 2003). But what causes the audience to devalue you here is the inference that you did something bad (De Jong et al., 2003), not your shame (although submissiveness and gaze avoidance can be aversive in its own right). In sum, the observed associations between shame and devaluation observed here and elsewhere more likely reflect the causal arrow from devaluation to shame hypothesized by the information threat theory.

The present study has limitations. It remains to be determined whether shame tracks devaluation precisely also when shame is elicited by real events and when shame is assayed with measures other than self-reported. Although there is substantial cultural dissimilarity between the United States and India (Muthukrishna et al., 2020; Shweder, 2003), one must also consider that the present study was run in English through MTurk in India (as well as the United States). Therefore, the Indian sample may not be representative of the general Indian population, wherein fewer than 1% has English as first language. Thus, it is possible that we effectively sampled less than the two cultures we sampled nominally, and this might account for the observed similarities between the United States and India. Further research with other populations and cultures can shed light on this point.

Ambiguity in the data too is a limitation. Although the data are consistent with our aim to measure the outputs of two different psychological systems (shame, social-evaluative psychology), the data are also consistent with the possibility that the ratings of shame and the ratings of devaluation were in fact delivered by one and the same psychological system, if, for example, participants in both conditions interpreted the prompts as soliciting ratings of the wrongness or badness of various scenarios. If so, the results would more modestly indicate that people generally agree with one another about how unacceptable each of various actions is when the actions are, as in the present research, relatively uncontroversial—note that there is previous evidence for this type of semantic consensus (Moore et al., 1999). Relatedly, whereas the data are consistent with the information threat hypothesis that shame in the actor tracks the degree to which *other individuals* disvalue the relevant disgraceful action (or those who take that action), the fact that our data showed within- and between-culture agreement about how disgraceful various actions are suggests that we cannot rule out the alternative hypothesis that shame in the actor tracks the degree to which *the actor herself* disvalue the relevant disgraceful action (or those who take that action)^1^. To show that shame in the actor tracks the values of audiences rather than the values of the actor herself, one needs a different method; one wherein the values of the actor and the values of the audience are systematically decoupled. This ambiguity is problematic: If shame in our participants truly tracked the degree to which *our participants themselves* disvalue a disgraceful action, that would offer support to alternative theories of shame—for example, attributional theories of shame, wherein shame is triggered when the actor falls short of her own standards *in her own estimation* (Tangney et al., 2007; Tracy & Robins, 2004). Despite this ambiguity in our data, various lines of evidence suggest that, ultimately, the shame system tracks the evaluations *of others* and, more specifically, the likelihood and cost of being devalued by others, as the information threat theory posits. First, autobiographical accounts indicate that being a victim of torture (Shapiro, 2003) or rape (Notman & Nadelson, 1976) elicits shame. In particular, shame is prevalent in victims of subjugation, where “on a rational plane, there should not have been much to be ashamed of” (Levi, 1989, p. 77). That is, devaluation by others triggers one's shame even one knows that one's actions are morally unimpeachable. There is experimental evidence for this effect: Indications of being devalued (vs. valued) by others increase shame in the actor, *even among those individuals who know they have acted virtuously with respect to those others* (Robertson et al., 2018). Second, experimental data indicate that the publicity (vs. privacy) of a disgraceful or potentially disgraceful act by the actor increases shame in the actor (Robertson et al., 2018; Smith et al., 2002). Thus, shame is sensitive to what other people know about the actor, because in those experiments what the actor knows about herself is constant across the spectrum of privacy-publicity. Third, experimental data indicate that the same harm imposed unintentionally on another individual elicits more shame in the harming actor when the other is one's likeable employee than when the other is one's tyrannical boss (Landers et al., forthcoming). Thus, shame is sensitive to the estimated severity of the devaluation from others, because in those experiments the transgression is the same across victim types. Nevertheless, more research is needed to address the ambiguity in the present data.

In conclusion, anticipatory feelings of shame appear to be precisely quantitatively matched to the magnitude of the devaluative threat on an event-by-event basis. This is consistent with the information threat theory, because a precise match is a key element in a broader architecture that is improbably well suited to balance the competing demands of effectiveness and economy and, more generally, to defend against the threat of devaluation (Landers et al., in press; Sznycer, 2010). Because shame leads to feelings of inferiority and worthlessness, and to evasions and aggression, shame has been deemed maladaptive, pathogenic, and ugly (Tangney, Wagner and Gramzow, 1992; Tangney et al., 1996; but see Tangney et al., 2014). However, shame evinces precision, functionality, and cross-cultural regularity when its operation is evaluated against the demands posed by information-triggered devaluation. From this adaptationist standpoint, the shame system is elegantly engineered to forego socially costly choices and minimize the costs of devaluation.

## Supplemental Material

sj-docx-1-evp-10.1177_14747049231203394 - Supplemental material for The Shame System Operates With High Precision Click here for additional data file. Supplemental material, sj-docx-1-evp-10.1177_14747049231203394 for The Shame System Operates With High Precision by Alexie Leroux, Sébastien Hétu and Daniel Sznycer in Evolutionary Psychology

## Notes

1. We thank an anonymous reviewer for pointing this out.
