# Supplementary material for: The Shame System Operates With High Precision
Source: Evol Psychol. 2023 Sep 28;21(3):14747049231203394. doi: 10.1177/14747049231203394 (PMC10540588; doi:10.1177/14747049231203394)
Supplement: sj-docx-1-evp-10.1177_14747049231203394 - Supplemental material for The Shame System Operates With High Precision [file sj-docx-1-evp-10.1177_14747049231203394.docx]

**Supplementary information**

**The shame system operates with high precision**

Table S1. Shame condition: Scenarios by disgracefulness set.

| **Appropriate set** | **Mildly Disgraceful set** | **Disgraceful set** |
| --- | --- | --- |
| I am in the bike lane cycling. | I am on the city sidewalk cycling. | I am on the highway cycling. |
| I am at the bar flirting. | I am at the post office flirting. | I am at the Doctor’s office flirting. |
| I am at a wedding kissing. | I am on the bus kissing. | I am in the Doctor’s office kissing. |
| I am at a choir performance singing. | I am on a city sidewalk singing. | I am in a hospital singing. |
| I am at a real estate’s office bargaining. | I am in a taxi bargaining. | I am at a church bargaining. |
| I am in my room swearing. | I am at the convenience store swearing. | I am at the daycare with children swearing. |
| I am on my couch sleeping. | I am on a park bench sleeping. | I am at a class lecture sleeping. |
| I am at a coffee bar talking. | I am in a dance class talking. | I am in the movie theatre talking. |
| I am at a rock concert yelling. | I am on the metro yelling. | I am in the library yelling. |
| I am in my bedroom yawning. | I am at the coffee bar yawning. | I am at a meeting with your boss yawning. |
| I am at a symphony concert applauding. | I am at the hotel lobby applauding. | I am at a funeral applauding. |
| I am at a wedding taking photos. | I am at a restaurant taking photos. | I am at a funeral taking photos. |
| I am in the dining hall eating a sandwich. | I am on the bus eating a sandwich. | I am at a funeral eating a sandwich. |
| I am in the living room hugging. | I am in the park hugging. | I am at an office meeting hugging. |
| I am at the bar laughing out loud. | I am on the bus laughing out loud. | I am at a funeral laughing out loud. |
| I am at home talking on my cellphone. | I am at the post office talking on my cellphone. | I am in the movie theatre talking on my cellphone. |
| I am in my room texting. | I am at the gym texting. | I am at church texting. |
| I am on the beach sunbathing. | I am on campus sunbathing. | I am at a funeral sunbathing. |
| I am in the bathroom putting on lipstick. | I am in the bank putting on lipstick. | I am in the church putting on lipstick. |
| I am in the public restroom breastfeeding my baby. | I am in the park breastfeeding my baby. | I am in the hotel lobby breastfeeding my baby. |
| I am in the living room chewing gum. | I am at the theatre chewing gum. | I am on a date chewing gum. |
| I am on the train listening to music on my headphones. | I am at the bar listening to music on my headphones. | I am at a class lecture listening to music on my headphones. |
| I am in the bathroom brushing my teeth. | I am in the public restroom brushing my teeth. | I am in the restaurant brushing my teeth. |
| I am in the art studio painting. | I am on the street corner painting. | I am in the restaurant painting. |
| I am at the library reading a book. | I am at the supermarket reading a book. | I am at the church reading a book. |

Table S2. Devaluation condition: Scenarios by disgracefulness set.

| **Appropriate set** | **Mildly Disgraceful set** | **Disgraceful set** |
| --- | --- | --- |
| Jacob is in the bike lane cycling. | Jacob is on the city sidewalk cycling. | Jacob is on the highway cycling. |
| Thomas is at the bar flirting. | Thomas is at the post office flirting. | Thomas is at the Doctor’s office flirting. |
| Emma is at a wedding kissing. | Emma is on the bus kissing. | Emma is in the Doctor’s office kissing. |
| Sarah is at a choir performance singing. | Sarah is on a city sidewalk singing. | Sarah is in a hospital singing. |
| Claire is at a real estate’s office bargaining. | Claire is in a taxi bargaining. | Claire is at a church bargaining. |
| Graham is in his room swearing. | Graham is at the convenience store swearing. | Graham is at the daycare with children swearing. |
| Kristen is on her couch sleeping. | Kristen is on a park bench sleeping. | Kristen is at a class lecture sleeping. |
| Mia is at a coffee bar talking. | Mia is in a dance class talking. | Mia is in the movie theatre talking. |
| Steven is at a rock concert yelling. | Steven is on the metro yelling. | Steven is in the library yelling. |
| Michael is in his bedroom yawning. | Michael is at the coffee bar yawning. | Michael is at a meeting with his boss yawning. |
| John is at a symphony concert applauding. | John is at the hotel lobby applauding. | John at a funeral applauding. |
| Isabella is at a wedding taking photos. | Isabella is at a restaurant taking photos. | Isabella is at a funeral taking photos. |
| Bob is in the dining hall eating a sandwich. | Bob is on the bus eating a sandwich. | Bob is at a funeral eating a sandwich. |
| David is in the living room hugging. | David is in the park hugging. | David is at an office meeting hugging. |
| Clark is at the bar laughing out loud. | Clark is on the bus laughing out loud. | Clark is at a funeral laughing out loud. |
| Josh is at home talking on my cellphone. | Josh is at the post office talking on my cellphone. | Josh is in the movie theatre talking on my cellphone. |
| Heather is in her room texting. | Heather is at the gym texting. | Heather is at church texting. |
| Hannah is on the beach sunbathing. | Hannah is on campus sunbathing. | Hannah is at a funeral sunbathing. |
| Sophia is in the bathroom putting on lipstick. | Sophia is in the bank putting on lipstick. | Sophia is in the church putting on lipstick. |
| Lisa is in the public restroom breastfeeding her baby. | Lisa is in the park breastfeeding her baby. | Lisa is in the hotel lobby breastfeeding her baby. |
| John is in the living room chewing gum. | John is at the theatre chewing gum. | John is on a date chewing gum. |
| Liam is on the train listening to music on his headphones. | Liam is at the bar listening to music on his headphones. | Liam is at a class lecture listening to music on his headphones. |
| Emily is in the bathroom brushing her teeth. | Emily is in the public restroom brushing her teeth. | Emily is in the restaurant brushing her teeth. |
| Elizabeth is in the art studio painting. | Elizabeth is on the street corner painting. | Elizabeth is in the restaurant painting. |
| Cindy is at the library reading a book. | Cindy is at the supermarket reading a book. | Cindy is at the church reading a book. |

Table S3. Ratings of shame and devaluation by scenario and country.

| Set | Scenario | United States | | India | |
| --- | --- | --- | --- | --- | --- |
|  |  | Shame | Devaluation | Shame | Devaluation |
| A | I am in my bedroom yawning. / Michael is in his bedroom yawning. | 1.32 (1.13) | 1.85 (1.60) | 4.31 (2.09) | 4.39 (2.19) |
| A | I am in the art studio painting. / Elizabeth is in the art studio painting. | 1.35 (1.09) | 1.74 (1.37) | 4.19 (2.10) | 4.19 (2.08) |
| A | I am on my couch sleeping. / Kristen is on her couch sleeping. | 1.38 (1.09) | 1.95 (1.70) | 4.12 (2.06) | 4.08 (2.07) |
| A | I am in my room texting. / Heather is in her room texting. | 1.41 (1.04) | 1.79 (1.40) | 4.08 (1.74) | 4.14 (2.13) |
| A | I am at home talking on my cellphone. / Josh is at home talking on my cellphone. | 1.41 (1.12) | 1.74 (1.39) | 3.69 (1.99) | 4.17 (2.10) |
| A | I am at the library reading a book. / Cindy is at the library reading a book. | 1.41 (1.21) | 1.64 (1.18) | 3.85 (2.33) | 4.19 (2.34) |
| A | I am in the living room hugging. / David is in the living room hugging. | 1.41 (1.23) | 1.77 (1.48) | 4.23 (1.80) | 4.28 (2.20) |
| A | I am on the train listening to music on my headphones. / Liam is on the train listening to music on his headphones. | 1.43 (1.17) | 1.90 (1.74) | 4.38 (2.12) | 4.11 (2.20) |
| A | I am in the bathroom brushing my teeth. / Emily is in the bathroom brushing her teeth. | 1.49 (1.19) | 1.69 (1.40) | 3.54 (2.04) | 3.83 (2.01) |
| A | I am in the dining hall eating a sandwich. / Bob is in the dining hall eating a sandwich. | 1.51 (1.19) | 1.95 (1.76) | 3.85 (1.85) | 4.19 (2.16) |
| A | I am at a symphony concert applauding. / John is at a symphony concert applauding. | 1.54 (1.17) | 2.08 (1.66) | 4.31 (1.44) | 4.61 (2.05) |
| A | I am in the living room chewing gum. / John is in the living room chewing gum. | 1.54 (1.35) | 1.82 (1.63) | 4.08 (2.01) | 4.08 (1.95) |
| A | I am in the bathroom putting on lipstick. / Sophia is in the bathroom putting on lipstick. | 1.57 (1.34) | 1.67 (1.26) | 3.65 (1.79) | 4.31 (2.09) |
| A | I am at a coffee bar talking. / Mia is at a coffee bar talking. | 1.59 (1.21) | 2.05 (1.73) | 3.96 (1.90) | 3.94 (1.91) |
| A | I am at a wedding taking photos. / Isabella is at a wedding taking photos. | 1.70 (1.43) | 1.67 (1.34) | 3.62 (2. 08) | 4.17 (2.05) |
| A | I am in the bike lane cycling. / Jacob is in the bike lane cycling. | 1.70 (1.58) | 1.82 (1.52) | 4.12 (1.86) | 4.75 (1.93) |
| A | I am on the beach sunbathing. / Hannah is on the beach sunbathing. | 1.84 (1.59) | 1.90 (1.39) | 4.58 (2.02) | 4.31 (2.15) |
| M | I am at a coffee bar yawning. / Michael is at a coffee bar yawning. | 1.92 (1.38) | 2.20 (1.64) | 4.50 (1.36) | 4.47 (2.00) |
| M | I am in the park hugging. / David is in the park hugging. | 1.95 (1.35) | 2.46 (1.86) | 4.54 (1.68) | 4.39 (1.96) |
| A | I am in the public restroom breastfeeding my baby. / Lisa is in the public restroom breastfeeding her baby. | 1.95 (1.45) | 2.08 (1.74) | 4.15 (1.85) | 4.08 (2.09) |
| M | I am at the theatre chewing gum. / John is at the theatre chewing gum. | 1.97 (1.62) | 2.10 (1.33) | 4.15 (2.13) | 4.28 (1.88) |
| M | I am at the gym texting. / Heather is at the gym texting. | 2.00 (1.45) | 2.67 (1.84) | 4.73 (1.66) | 4.67 (2.00) |
| A | I am at a choir performance singing. / Sarah is at a choir performance singing. | 2.00 (1.58) | 1.46 (1.12) | 3.77 (1.77) | 4.17 (2.02) |
| A | I am in my room swearing. / Graham is in his room swearing. | 2.00 (1.68) | 2.82 (1.90) | 4.46 (1.79) | 4.33 (1.87) |
| A | I am at a rock concert yelling. Steven is at a rock concert yelling. | 2.19 (1.71) | 2.54 (1.93) | 4.69 (1.35) | 4.22 (1.73) |
| A | I am at the bar laughing out loud. / Clark is at the bar laughing out loud. | 2.24 (1.75) | 2.10 (1.57) | 4.58 (1.81) | 4.56 (1.95) |
| M | I am at the supermarket reading a book. / Cindy is at the supermarket reading a book. | 2.38 (1.51) | 3.15 (1.88) | 4.38 (1.67) | 4.56 (1.68) |
| A | I am at a real estate’s office bargaining. / Claire is at a real estate’s office bargaining. | 2.41 (1.61) | 1.90 (1.50) | 4.35 (1.47) | 4.47 (1.95) |
| A | I am at the bar flirting. / Thomas is at the bar flirting. | 2.51 (1.68) | 2.46 (1.70) | 4.65 (1.67) | 4.44 (1.89) |
| M | I am at the bar listening to music on my headphones. / Liam is at the bar listening to music on his headphones. | 2.54 (1.73) | 2.28 (1.60) | 4.50 (1.77) | 4.03 (2.11) |
| A | I am at a wedding kissing. / Emma is at a wedding kissing. | 2.84 (1.82) | 2.33 (1.63) | 4.65 (1.79) | 4.22 (2.01) |
| M | I am on the bus eating a sandwich. / Bob is on the bus eating a sandwich. | 2.86 (1.73) | 2.33 (1.54) | 4.46 (1.73) | 4.19 (1.97) |
| M | I am on the street corner painting. / Elizabeth is on the street corner painting. | 2.92 (1.86) | 2.49 (1.68) | 4.50 (1.48) | 4.22 (2.03) |
| M | I am at the hotel lobby applauding. / John is at the hotel lobby applauding. | 3.03 (1.71) | 3.18 (1.70) | 4.85 (1.19) | 4.56 (1.90) |
| M | I am at a restaurant taking photos. / Isabella is at a restaurant taking photos. | 3.05 (1.67) | 2.38 (1.53) | 4.04 (1.86) | 4.33 (1.94) |
| D | I am on a date chewing gum. / John is on a date chewing gum. | 3.08 (1.99) | 2.92 (1.85) | 4.50 (2.04) | 4.36 (1.97) |
| M | I am at the post office talking on my cellphone. / Josh is at the post office talking on my cellphone. | 3.22 (1.89) | 3.36 (1.65) | 4.31 (1.82) | 4.17 (2.08) |
| M | I am in a taxi bargaining. / Claire is in a taxi bargaining. | 3.27 (1.85) | 3.54 (1.76) | 4.23 (1.80) | 4.33 (2.01) |
| M | I am on the bus laughing out loud. / Clark is on the bus laughing out loud. | 3.27 (1.94) | 2.72 (1.73) | 4.73 (1.59) | 4.53 (1.95) |
| M | I am at the post office flirting. / Thomas is at the post office flirting. | 3.30 (1.71) | 3.38 (1.77) | 4.65 (1.60) | 4.89 (1.53) |
| M | I am in the park breastfeeding my baby. / Lisa is in the park breastfeeding her baby. | 3.30 (2.12) | 3.00 (2.15) | 4.27 (1.89) | 4.36 (2.10) |
| M | I am on a city sidewalk singing. / Sarah is on a city sidewalk singing. | 3.30 (2.20) | 2.59 (1.67) | 4.35 (1.47) | 4.19 (1.95) |
| M | I am on the city sidewalk cycling. / Jacob is on the city sidewalk cycling. | 3.41 (2.07) | 3.21 (1.66) | 4.04 (1.75) | 4.39 (2.00) |
| M | I am in a dance class talking. / Mia is in a dance class talking. | 3.49 (1.95) | 3.00 (1.91) | 4.38 (1.44) | 4.72 (1.63) |
| M | I am in the public restroom brushing my teeth. / Emily is in the public restroom brushing her teeth. | 3.49 (2.01) | 3.31 (2.00) | 4.92 (1.52) | 4.89 (1.65) |
| M | I am at the bank putting on lipstick. / Sophia is at the bank putting on lipstick. | 3.49 (2.10) | 3.05 (1.77) | 4.50 (1.21) | 4.36 (1.81) |
| D | I am at the church reading a book. / Cindy is at the church reading a book. | 3.65 (2.20) | 3.33 (2.17) | 4.38 (1.90) | 4.72 (1.89) |
| D | I am on the highway cycling. / Jacob is on the highway cycling. | 3.68 (2.15) | 4.44 (2.07) | 4.04 (1.93) | 4.47 (1.93) |
| D | I am at an office meeting hugging. / David is at an office meeting hugging. | 3.78 (1.87) | 3.46 (1.86) | 4.69 (1.41) | 4.69 (1.75) |
| D | I am in the church putting on lipstick. / Sophia is in the church putting on lipstick. | 3.89 (2.14) | 3.95 (2.22) | 5.15 (1.35) | 5.22 (1.48) |
| M | I am on campus sunbathing. / Hannah is on campus sunbathing. | 3.92 (2.20) | 4.03 (1.88) | 5.31 (1.12) | 4.94 (1.64) |
| D | I am in a hospital singing. / Sarah is in a hospital singing. | 4.00 (2.05) | 3.36 (1.65) | 5.19 (1.50) | 5.03 (1.71) |
| D | I am at the Doctor’s office flirting. / Thomas is at the Doctor’s office flirting. | 4.03 (1.98) | 3.92 (1.82) | 4.92 (1.49) | 5.19 (1.64) |
| M | I am on the bus kissing. / Emma is on the bus kissing. | 4.05 (2.01) | 3.46 (1.73) | 5.42 (1.60) | 4.86 (1.68) |
| D | I am in the restaurant painting. / Elizabeth is in the restaurant painting. | 4.19 (2.14) | 3.36 (1.81) | 4.62 (1.28) | 4.75 (1.90) |
| M | I am on a park bench sleeping. / Kristen is on a park bench sleeping. | 4.27 (2.09) | 3.49 (1.90) | 4.81 (1.44) | 4.50 (1.56) |
| D | I am at a meeting with your boss yawning. / Michael is at a meeting with his boss yawning. | 4.35 (1.95) | 3.56 (1.81) | 4.73 (1.61) | 4.69 (1.70) |
| D | I am at a funeral taking photos. / Isabella is at a funeral taking photos. | 4.59 (2.07) | 3.67 (1.72) | 5.27 (2.22) | 4.94 (1.49) |
| D | I am in the hotel lobby breastfeeding my baby. / Lisa is in the hotel lobby breastfeeding her baby. | 4.62 (2.00) | 4.15 (1.98) | 4.50 (1.79) | 4.42 (1.96) |
| D | I am at a church bargaining. / Claire is at a church bargaining. | 4.65 (2.15) | 4.41 (2.09) | 4.85 (1.51) | 5.03 (1.56) |
| D | I am in the Doctor’s office kissing. / Emma is in the Doctor’s office kissing. | 4.70 (1.81) | 4.38 (1.91) | 5.12 (1.31) | 5.44 (1.38) |
| D | I am at a class lecture listening to music on my headphones. / Liam is at a class lecture listening to music on his headphones. | 4.78 (1.87) | 4.67 (1.80) | 4.73 (1.56) | 5.11 (1.43) |
| M | I am at the convenience store swearing. / Graham is at the convenience store swearing. | 4.81 (2.00) | 4.72 (1.73) | 4.65 (1.20) | 4.61 (1.82) |
| M | I am on the metro yelling. / Steven is on the metro yelling. | 4.81 (2.12) | 5.05 (1.38) | 4.96 (1.37) | 4.61 (1.66) |
| D | I am in the movie theatre talking. / Mia is in the movie theatre talking. | 4.89 (1.66) | 5.23 (1.75) | 4.38 (1.86) | 4.67 (1.88) |
| D | I am at church texting. / Heather is at church texting. | 4.92 (2.09) | 4.13 (1.91) | 4.85 (1.40) | 4.94 (1.64) |
| D | I am in the restaurant brushing my teeth. / Emily is in the restaurant brushing her teeth. | 5.11 (1.94) | 4.69 (2.09) | 5.23 (1.48) | 4.69 (1.92) |
| D | I am at a class lecture sleeping. / Kristen is at a class lecture sleeping. | 5.54 (1.57) | 4.28 (1.96) | 5.35 (1.23) | 5.28 (1.50) |
| D | I am at a funeral eating a sandwich. / Bob is at a funeral eating a sandwich. | 5.70 (1.71) | 4.95 (1.86) | 5.23 (1.24) | 5.28 (1.39) |
| D | I am at a funeral applauding. / John at a funeral applauding. | 5.86 (1.72) | 4.74 (1.90) | 5.15 (1.38) | 5.42 (1.32) |
| D | I am at a funeral laughing out loud. / Clark is at a funeral laughing out loud. | 5.97 (1.40) | 5.18 (1.48) | 5.69 (1.16) | 5.50 (1.13) |
| D | I am in the movie theatre talking on my cellphone. / Josh is in the movie theatre talking on my cellphone. | 5.97 (1.61) | 5.49 (1.76) | 4.42 (1.47) | 5.00 (1.62) |
| D | I am in the library yelling. / Steven is in the library yelling. | 6.08 (1.36) | 5.67 (1.54) | 4.88 (1.42) | 5.11 (1.60) |
| D | I am at the daycare with children swearing. Graham is at the daycare with children swearing. | 6.16 (1.42) | 5.64 (1.66) | 4.54 (1.39) | 4.69 (1.75) |
| D | I am at a funeral sunbathing. / Hannah is at a funeral sunbathing. | 6.62 (0.92) | 6.08 (1.56) | 5.35 (1.32) | 5.61 (1.55) |

A: Scenario in the Appropriate set, M: Scenario in the Mildly Disgraceful set, D: scenario in the Disgraceful set. Scenarios are displayed from lowest to highest mean shame ratings in the United States
